# Enteric pathogens through life stages

**DOI:** 10.3389/fcimb.2012.00114

**Published:** 2012-08-25

**Authors:** Glynis Kolling, Martin Wu, Richard L. Guerrant

**Affiliations:** ^1^Department of Internal Medicine, Division of Infectious Diseases and International Health, Center for Global Health, University of VirginiaCharlottesville, VA, USA; ^2^Department of Biology, University of VirginiaCharlottesville, VA, USA

**Keywords:** enteric pathogen, intestinal microbiota, malnutrition, diarrhea, age distribution

## Abstract

Enteric infections and diarrheal diseases constitute pervasive health burdens throughout the world, with rates being highest at the two ends of life. During the first 2–3 years of life, much of the disease burden may be attributed to infection with enteric pathogens including Salmonella, rotavirus, and many other bacterial, viral, and protozoan organisms; however, infections due to *Clostridium difficile* exhibit steady increases with age. Still others, like *Campylobacter* infections in industrialized settings are high in early life (<2 years old) and increase again in early adulthood (called the “second weaning” by some). The reasons for these differences undoubtedly reside in part in pathogen differences; however, host factors including the commensal intestinal microbial communities, immune responses (innate and acquired), and age-dependant shifts likely play important roles. Interplay of these factors is illustrated by studies examining changes in human gut microbiota with inflammatory bowel disease and irritable bowel syndrome. Recent gut microbial surveys have indicated dramatic shifts in gut microbial population structure from infants to young adults to the elders. An understanding of the evolution of these factors and their interactions (e.g., how does gut microbiota modulate the “inflamm-aging” process or vice versa) through the human life “cycle” will be important in better addressing and controlling these enteric infections and their consequences for both quality and quantity of life (often assessed as disability adjusted life-years or “DALYs”).

## Defining enteric “disease”

Defining “pathogens” first requires defining “disease.” We propose defining enteric disease as far more than simply passage of unformed stools (i.e., “diarrhea”) from one end of a very long and functionally critical “tube” to sustaining healthy life. Therefore, a broader definition of enteric disease would include: ≥3 or more unformed stools per day and any documented intestinal infection associated with disrupted intestinal absorptive and/or barrier function. This may impair growth in young children or cognitive function. Implicit in this more comprehensive concept of enteric disease is an urgent need for improved biomarkers of impaired intestinal absorptive and barrier function.

Thus, an enteric pathogen may be classified as any microbe that is able to cause enteric “disease” as defined above. Microbial pathogenesis can involve direct invasion, signals triggering host inflammation or other changes, or secreted factors that damage the host directly (e.g., toxins), indirectly (e.g., microbial competition), and/or by exploiting other environmental host-associated factors to thrive.

## Host locale of enteric disease

**Figure 1:**
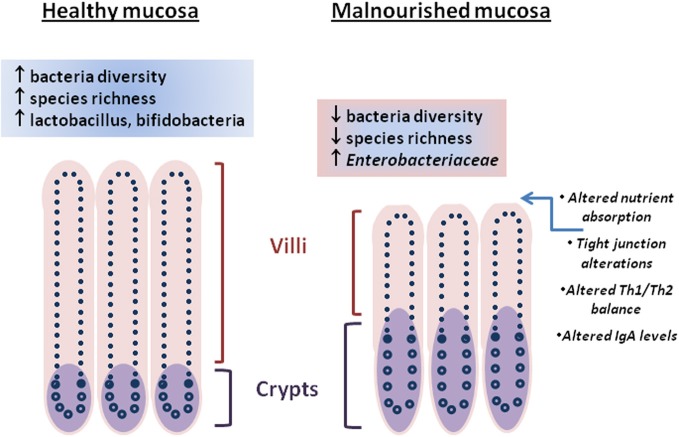
**Differences between “healthy” and malnourished intestinal mucosa are represented by architectural changes (villi height/crypt depth, crypt hypertrophy), molecular changes (tight junction alteration, nutrient absorption), immune changes, and the intestinal microbiota.** Cross-talk between the intestinal microbiota and intestinal mucosa through metabolites is unclear.

The intestinal epithelium is a heterogeneous mixture of cells poised to respond to the closely positioned microbiota and luminal contents. The tight junctions that exist between intestinal epithelial cells provide a maintained barrier (Marchiando et al., 2010) that when breached by enteric pathogen or toxin can lead to leakage of luminal contents into the underlying lamina propria where immune cells reside and potentially instigate deleterious inflammatory and other physiological responses leading to diarrhea or disrupted absorptive function (Su et al., 2009). Conversely, nutritional status, intestinal microbiota, and stress have been shown to play roles in maintaining barrier function (Brewster et al., 1997; Yang et al., 2006; Zareie et al., 2006). Perturbations of nutritional status and alteration of the intestinal microbiota is evident in persistent malnutrition and enteric infections (Mondal et al., 2012). Changes in the intestinal architecture during a malnourished state include blunted villi, crypt hypertrophy, and altered levels of intraepithelial lymphocytes leading to impaired immune responses, nutrient absorption (Figure 1) and ultimately decline in early childhood growth (Guerrant et al., 2008; Moore et al., 2010).

Effects of intestinal flora on the brain and enteric nervous system are now being elucidated. Early childhood enteric disease and malnutrition have been linked with impaired cognitive development (Lorntz et al., 2006; Laus et al., 2011). The profound effect of childhood malnutrition and enteric disease on cognitive function ultimately affects individual productivity in life; moreover, stunting early in life may increase risk for later obesity and possibly other chronic diseases (Guerrant et al., 2008; Victora et al., 2008). Factors such as *APOE4* may affect enteric disease and cognitive function. The *APOE4* allele in Brazilian children has been correlated with lower diarrhea burdens, and improved cognitive performance (Oria et al., 2005); cellular effects of APOE in the intestine and brain are under study (Vitek et al., 2009; Azevedo et al., 2012). While *APOE4* provides protection to children with enteric disease, it is also linked with the neurodegenerative, Alzheimer's disease (Strittmatter et al., 1993). Currently, no apparent links appear to exist between *APOE4* and malnutrition in the elderly (Matera et al., 2010); furthermore, limited studies have found associations between cognitive impairment and malnutrition or infections in the elderly, although causality remains unclear (Orsitto et al., 2009; Fagerstrom et al., 2011). The long-term impact of enteric infections in children is a subject of intense research; however, parallel studies in elders are lacking. Increasingly, research addressing the connection(s) between intestine, microbiota, and brain (Grenham et al., 2011) should illuminate host-microbe processes.

## Life stages, incidence, pathogens

**Figure 2:**
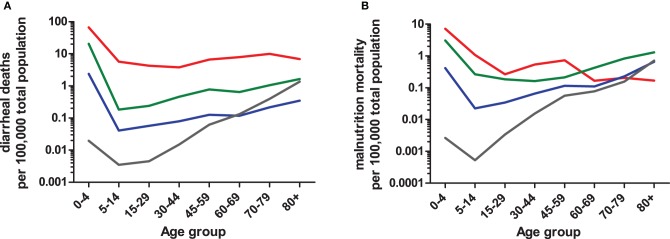
**Age-dependent mortality rates due to diarrhea (A) or protein-energy malnutrition (B) based on 2008 WHO data.** Numbers of diarrheal or malnutrition from the Global Burden of Disease in 2008 cases were compared to the total population sum of each WHO region (2012). WHO regions were grouped into four categories (i.e., low, low-middle, upper-middle, and upper) based on Gross National Income (GNI) brackets established by the World Bank (2012). The GNI income bracket most represented within a WHO region was used to combine data. For example, the Region of Americas (AMRB) consists of 26 countries, the majority of which (*n* = 18) are classified as upper-middle income countries and were therefore included in the upper-middle income data. The low-income (red line) category consists of African Region (AFR D and E) and South East Asian Region (SEAR D); low-middle (green line) consists of Region of the Americas (AMR D), Eastern Mediterranean Region (EMR D), Western Pacific Region (WPR B), and SEAR B; upper-middle (blue line) consists of AMR B, EMR B, and European Region (EURB and C); upper (grey line) consists of AMR A, EUR A, and WPR A. The mortality rate data are limited by the use of all-age population data, so actual age-dependent rates may differ.

By expanding the definition of enteric disease, the role of the host:pathogen (H:P) balance throughout life stages can be addressed in several contexts. Globally, the highest rates of diarrhea mortality tend to occur during early and late-life stages (Figure 2A; WHO data); however, rates in countries with the lowest GNI remain high throughout life. The age-associated trend is evident in cases of salmonellosis with high indices in infants and elders ≥65 (Trevejo et al., 2003). *E. coli* infections impacting early and late life stages are pathogen dependent; furthermore, diarrheagenic *E. coli* segregate geographically (Qadri et al., 2005; Ochoa et al., 2009; Snedeker et al., 2009; Opintan et al., 2010). *Cryptosporidium* may have its greatest impact in children <1yo or in already stunted children, (Checkley et al., 1997, 1998, 2008; Putignani and Menichella, 2010) rotaviruses in the first 2 years of life; *Campylobacter* in early childhood and young adulthood (the “second weaning”) (Ailes et al., 2008; da Silva et al., 2010; Soofi et al., 2011), then *C. difficile* infections clearly increasing steadily in both frequency and severity with increased age (Zilberberg et al., 2008). Given age-dependent changes in the incidence of enteric diseases, additional factors affecting the H:P balance include: intestinal microbiota, environment/exposure, and immune response.

## Age-dependent shifts in the intestinal microbiota

**Figure 3:**
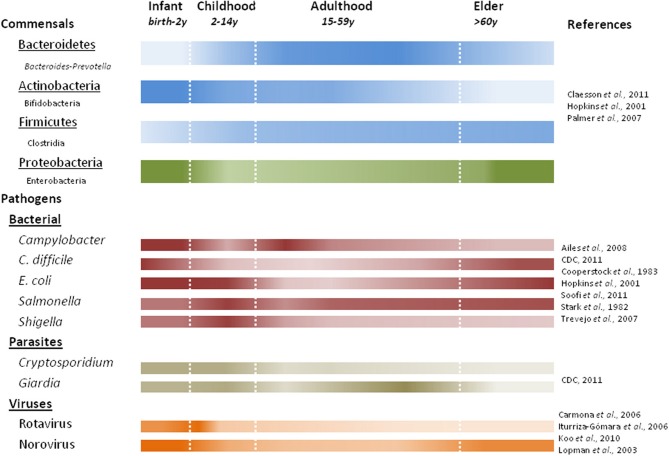
**Predominant commensal and pathogenic microbes associated with human life stages.** Color intensity of each bar correlates with presence or absence of commensal bacteria at the phylum (underlined) with examples listed below using noted references. Studies characterizing fecal microbiota prior to 2007 quantify the viable counts of bacteria, while later studies use DNA based methods.

The gut microbiota play important roles in maintaining the human health, including energy and nutrient extraction (Deguchi et al., 1985; Cummings and Macfarlane, 1991, 1997; Turnbaugh and Gordon, 2009), host immune system modulation and protection against pathogens (van der Waaij et al., 1971; Rolfe, 1997). Understanding of the diversity of human gut microbiota has improved dramatically in recent years largely due to culture-independent 16S rRNA based surveys. Recent studies have shown large interpersonal variations in the gut microbiota as a function of age (Figure 3), health status and diet (Dethlefsen et al., 2006; Ley et al., 2006; Mueller et al., 2006; Woodmansey, 2007; Tannock, 2008; O'Toole and Claesson, 2010; Claesson et al., 2011; Wu et al., 2011). Nevertheless, population based studies show three “enterotypes” with potential diagnostic value (Arumugam et al., 2011). The enterotypes are based on the preponderance of three genera: *Bacteroides*, *Prevotella*, and *Ruminococcus*. It is likely that additional enterotypes will surface when other health-related factors are considered (De et al., 2010; Monira et al., 2011; Wu et al., 2011).

The gut microbiota during early **infancy** is relatively simple but highly dynamic (Favier et al., 2002; Palmer et al., 2007). Incidental exposures (e.g., maternal microbiota, breastfeeding vs. formula) play a major role in seeding the neonatal gut (Bennet et al., 1991; Mandar and Mikelsaar, 1996; Penders et al., 2006; Adlerberth and Wold, 2009). As a result, the progression of early colonization is often chaotic and variable (Palmer et al., 2007). Facultative anaerobes, like *E. coli* (Hopkins et al., 2001; Salminen and Isolauri, 2006; Mariat et al., 2009) and Gram-negative obligate anaerobes (e.g., *Bacteroides*-*Prevotella*) dominate early followed by a predominance of bifidobacteria by three months of age (Mah et al., 2007; Mariat et al., 2009). Infection with *Salmonella* and pathogenic *E. coli* pose the greatest risks in early infancy when facultative anaerobes are predominant. Over the first year, through a series of successions and replacements, the infant microbial communities become more similar to one another, converging toward a more stable adult-like profile (Favier et al., 2002; Palmer et al., 2007), characterized by a preponderance of *Bacteroidetes* and *Firmicutes*. In addition to the composition difference, quantitative PCR show total bacterial counts in infants to be nearly ten-fold lower than in adults and elders (Mariat et al., 2009).

Gut microbiota in young **adults** are dominated by *Bacteroidetes* and *Firmicutes* (making up approximately 95% of the microbiota) with smaller fractions of *Actinobacteria* and *Proteobacteria* (Ley et al., 2006; Andersson et al., 2008; Tap et al., 2009). The peak numbers and diversity in gut microbial composition is achieved near the end of adolescence. Still, each adult's gut appears to harbor a unique microbial community that remains relatively stable through adulthood (Franks et al., 1998; Zoetendal et al., 1998; Vanhoutte et al., 2004; Leser and Molbak, 2009). One recent study suggested that stability may last longer than expected, and that **aging** starts to affect the gut microbiota after 65 years of age (Biagi et al., 2010). In addition, decreased intestinal motility (Brocklehurst, 1972; Macfarlane et al., 1989), dietary changes (Flint et al., 2007), and “inflamm-aging” (a chronic low-grade inflammation in elders) (Franceschi, 2007; Guigoz et al., 2008) all affect homeostasis of the gut microbial ecosystem. Recent studies indicate dramatic shifts in the composition of gut commensals in elders. When elders were compared to young adults, a decrease in the relative abundance of *Bifidobacteria* and *Firmicutes* was observed, accompanied by a commensurate increase in *Bacteroides* and facultative anaerobes (Hopkins and Macfarlane, 2002; Mariat et al., 2009; Claesson et al., 2011), although large compositional variations were also found among elderly individuals and populations (Mueller et al., 2006; Claesson et al., 2011). The decline in beneficial bifidobacteria is one of the most marked changes in the aging gut, manifested in both microbial abundance and species diversity (Mitsuoka et al., 1974; Benno et al., 1992; Mitsuoka, 1992; He et al., 2001; Hopkins et al., 2001; Hopkins and Macfarlane, 2002). Such a population shift could increase the susceptibility of elders to *C. difficile* infections (CDI) (Woodmansey, 2007; Guigoz et al., 2008), as a similar but more pronounced composition change has been observed in CDI patients (Hopkins et al., 2001; Hopkins and Macfarlane, 2002). Furthermore, a stable gut microbiota is crucial in preventing *C. difficile* overgrowth. This is supported by studies showing significant reductions in the microbial diversity in patients with recurrent CDI (Chang et al., 2008), association of CDI onset with altered microbiota composition(s) before antibiotic treatment (De La Cochetiere et al., 2008), and prevention of primary or recurrent CDI through probiotic therapy or fecal transplantation, respectively (Hickson et al., 2007; Khoruts et al., 2010). *Bifidobacteria* and butyrate-producing bacteria (e.g., *Clostridium* clusters IV and XIVa) have been suggested to play important roles in providing colonization resistance against *C. difficile* (Fallani et al., 2006; Rousseau et al., 2011); however, more comprehensive studies are required to substantiate these claims (Gore et al., 2008).

In addition to CDI, changes in the gut microbial composition have been linked to childhood allergies and inflammatory bowel diseases (IBD). It has been proposed that the gut microbiota in infants modulates the mucosal immune response to environmental allergens (Tannock, 2007). Interestingly, differences in the bifidobacterial populations have been associated with atopic diseases and the allergic status of children (Sepp et al., 1997; Ouwehand et al., 2001; Gore et al., 2008; Hong et al., 2010). IBD, such as Crohn's disease and ulcerative colitis are usually diagnosed in adolescence or early adulthood. Several lines of evidence suggest a microbial etiology in the development of IBD (D'Haens et al., 1998; Sellon et al., 1998; Gionchetti et al., 2000; Shen et al., 2001; Sartor, 2004). Previous studies have led to the hypothesis that altered gut microflora, excluding a specific pathogen, contribute to the etiology of IBD. Loss of microbial diversity and richness, especially in members of clostridial cluster IV (e.g., *Faecalibacterium prausnitzii)*, was observed in Crohn's disease patients (Martinez-Medina et al., 2006; Scanlan et al., 2006; Frank et al., 2007; Sokol et al., 2008; Cucchiara et al., 2009). An increased *Bacteroidetes:Firmicutes* ratio and a predominance of opportunistic *Proteobacteria* have been reported in pediatric and adult IBD patients (Cucchiara et al., 2009). However, there is an ongoing debate on whether these changes are the cause or an effect of the disease (Sokol et al., 2008; Stecher and Hardt, 2008).

## Common themes of the microbiota

As already mentioned, a loss of species diversity and richness within the microbiota has been observed in IBD and CDI; in addition, based on their relative abundance, facultative anaerobes including members of *Proteobacteria* appear to thrive. Increased levels of *Proteobacteria* are also seen in malnourished children or in patients with celiac's disease (Bonventre, 1990; Monira et al., 2011). On a gross level, these findings also tend to occur with older age when this microbiota “structure” could alter other host functions (e.g., immune response). For example, the relative abundance of *Bacteroidetes* accounted for <15% of the intestinal community in 71% of malnourished Bangladeshi children compared to >40% relative abundance in 71% of healthy cases.

## Exposure and host responses

The environment plays a major role in acquisition of the pathogen; in many cases exposure to contaminated food and/or water are the main vehicles for pathogen transmission. Globally, countries in the low and low-middle income brackets face the highest rates of diarrhea-associated mortalities over much of the life cycle (Figure 2). The age-dependent mortality due to diarrhea evident at both ends of the life cycle has dramatic regional differences impacted in part by socio-economic category (e.g., developed vs. developing countries). Additional factors likely include polymicrobial infections, diet, host-microbiota relationship, waning maternal antibody and other regional specific co-morbidities. Indeed, this rationale could be applied to similar rates of diarrhea mortality among elders living in WHO regions falling into upper-middle and upper income categories in contrast to respective regional infant rates. In the case of the USA and Canada (i.e., AMRA), infection with *C. difficile* in healthcare or long-term care facility environments contribute to age-associated increases in mortality (Zilberberg et al., 2008). Depending on the setting, a patient may be treated with antibiotics that disrupt the “normal” microbiota resulting in susceptibility to opportunistic pathogens. Furthermore, prolonged stay within a hospital environment favors the transfer of nosocomial pathogens, in particular *C. difficile* (McFarland et al., 1989).

Conversely, exposure to pathogens in early infancy is greatest as children are weaned. In areas with high diarrhea burdens, both introduction of weaning foods and cessation of breastfeeding was associated with increased risk of dehydrating diarrhea (Guerrant et al., 1983; Fuchs et al., 1996). Consequently, increased malnutrition-based mortality is coincident with increased diarrhea mortality (Figure 2B). Adaptations in host immune responses and intestinal microbiota are crucial for the host to survive constant exposure to malnutrition or undernutrition and diarrhea disease throughout much of the life cycle, and these adaptations offer protection in later life when immune status wanes. In neonates, colonization and stabilization of intestinal microbial communities is intertwined with immune system development (Shulzhenko et al., 2011) with similar processes, albeit senescent in nature, occuring in elders (Biagi et al., 2010) suggesting age-dependant alterations of these systems are closely linked to pathogen resistance/susceptibility.

The innate immune system consists of frontline defenses to pathogens (Hooper and Macpherson, 2010) with parallel or subsequent responses by the adaptive arm of the immune system. Maturation and senescence of components in the adaptive immunity have been reviewed elsewhere (Adkins et al., 2004; Cancro et al., 2009). Antibody-mediated protection *in utero* and postpartum occurs through maternal, passive transfer and is cleared by 5 months of age; the age at which growth shortfalls often start (Victora et al., 2010). The impact of diet affects the host and subsequent adaptive responses; during malnutrition, both B- and T-cell responses are altered. In young mice and children, protein energy malnutrition decreases mucosal IgA responses potentially impacting the course of infection (Green and Heyworth, 1980; McGee and McMurray, 1988; Oberhelman et al., 1991). Conversely, patients with little to no IgA due to common variable immunodeficiency were more likely to be malnourished (Muscaritoli et al., 2001) suggesting important links between the immune system and nutritional status, which have been recently tied to the intestinal microbiota (Shulzhenko et al., 2011). The effect of malnutrition on T-cell responses during enteric infection have been examined in mice and humans with divergent results. In mice, tissue IFNγ increased in response to malnutrition and infection with *Cryptosporidium* or *Heligmosomoides* (Ing et al., 2000; Coutinho et al., 2008). Other *in vivo* studies show that protein-energy malnutrition alone ↑ serum IL-10 and ↓ IFNγ (Hillyer et al., 2007; Monk and Woodward, 2009) suggesting that infection further alters the immune response during malnutrition. Isolation and subsequent *in vitro* activation of CD4+ cells from malnourished or well-nourished, infected children, resulted in more IL-10 or IFNγ release characteristic of Th2 or Th1 bias, respectively (Rodriguez et al., 2005). The infecting pathogen(s) were not classified in this study, but samples from both gastrointestinal and respiratory infections were analyzed.

## Conclusions

The process of aging is associated with continuous changes in environmental exposures that subsequently affect the immune system and host-associated microbiota. Within the host's shifting landscape, infections by pathogens that cause enteric disease likely exploit these shifts with resultant initiation of pathogenesis and/or establishment of a mutualistic relationship with the host (e.g., carrier) leading to potential dissemination of the pathogen. There is still much to be learned about how life stages and pertubations shape the gut microbial population, and how these changes influence health and disease, and predominant enteric pathogens. For whatever population shifts we can associate with disease(s), it is necessary to demonstrate causality and not a mere effect or unrelated association with the diseases to fulfill Koch's postulates. Future insights into the interdependency between environment-host-microbe will be essential for development of novel therapeutic approaches that treat or prevent enteric disease. Understanding the roles of these factors within the host will be complementary to external approaches (e.g., sanitation, water treatment) for controlling or eradicating pathogen dissemination.

### Conflict of interest statement

The authors declare that the research was conducted in the absence of any commercial or financial relationships that could be construed as a potential conflict of interest.
